# Causal association evaluation of diabetes with Alzheimer's disease and genetic analysis of antidiabetic drugs against Alzheimer's disease

**DOI:** 10.1186/s13578-022-00768-9

**Published:** 2022-03-10

**Authors:** Lei Meng, Zhe Wang, Hong-Fang Ji, Liang Shen

**Affiliations:** 1 Institute of Biomedical Research, Shandong University of Technology, Zibo, Shandong People’s Republic of China; 2 Shandong Provincial Research Center for Bioinformatic Engineering and Technique, Zibo Key Laboratory of New Drug Development of Neurodegenerative Diseases, School of Life Sciences and Medicine, Shandong University of Technology, Zibo, Shandong People’s Republic of China

**Keywords:** Alzheimer’s disease, Diabetes, Causal association, Drug targets, Mendelian randomization

## Abstract

**Background:**

Despite accumulating epidemiological studies support that diabetes increases the risk of Alzheimer’s disease (AD), the causal associations between diabetes and AD remain inconclusive. The present study aimed to explore: i) whether diabetes is causally related to the increased risk of AD; ii) and if so, which diabetes-related physiological parameter is associated with AD; iii) why diabetes drugs can be used as candidates for the treatment of AD. Two-sample Mendelian randomization (2SMR) was employed to perform the analysis.

**Results:**

Firstly, the 2SMR analysis provided a suggestive association between genetically predicted type 1 diabetes (T1D) and a slightly increased AD risk (OR = 1.04, 95% CI = [1.01, 1.06]), and type 2 diabetes (T2D) showed a much stronger association with AD risk (OR = 1.34, 95% CI = [1.05, 1.70]). Secondly, further 2SMR analysis revealed that diabetes-related physiological parameters like fasting blood glucose and total cholesterol levels might have a detrimental role in the development of AD. Thirdly, we obtained 74 antidiabetic drugs and identified SNPs to proxy the targets of antidiabetic drugs. 2SMR analysis indicated the expression of three target genes, ETFDH, GANC, and MGAM, were associated with the increased risk of AD, while CPE could be a protective factor for AD. Besides, further PPI network found that GANC interacted with MGAM, and further interacted with CD33, a strong genetic locus related to AD.

**Conclusions:**

In conclusion, the present study provides evidence of a causal association between diabetes and increased risk of AD, and also useful genetic clues for drug development.

## Introduction

Alzheimer’s disease (AD) is known as the most common progressive neurodegenerative disease with an increasing prevalence worldwide. According to the World Alzheimer Report 2018 from Alzheimer’s Disease International, over 50 million people worldwide are suffering from dementia [1], and AD accounts for 60%-80% of all cases of dementia. With the aggravation of the disease, AD patients will show a series of clinical features, including progressive memory loss, gradual impairment of cognitive functions, behavioural and personality changes. Given the steadily increasing burdens on patients, families, and society, screening modifiable risk factors has been performed to reduce the risk of AD.

Diabetes, including type 1 diabetes (T1D), type 2 diabetes (T2D), and gestational diabetes, is a chronic metabolic disease with high blood glucose levels that can damage blood vessels and nerves and cause multiple serious complications. According to the International Diabetes Federation, 1 in 11 adults had diabetes (425 million people), and 12% of the global health expenditure was spent on diabetes in 2017 [2].

More recently, increasing attention has been paid to the associations of AD with several chronic disorders, among which diabetes has attracted much interest due to a series of pathogenic associations. For instance, in the past few decades, significant epidemiological evidence indicated that diabetes patients had an increased risk of developing AD by approximately 53% [3–5]. Besides, the mechanisms associated with diabetes, such as dysfunctional IR/PI3K/Akt signaling, increased inflammation, oxidative stress, and others, might accelerate the development of pathological events in AD [6, 7]. Moreover, a growing number of studies also supported the associations between AD and diabetes at the genetic level. A previous study has identified 395 SNPs to be shared the same risk allele for AD and T2D, suggesting common genetic aetiological risk factors between two disorders [8]. Correspondingly, inspired by the close association between two disorders, the studies of examining antidiabetic drugs against AD have increased tremendously. Excitedly, preliminary studies have indicated that many antidiabetic drugs, such as liraglutide, pioglitazone, lixisenatide, rosiglitazone, insulin, and exendin-4, exhibited therapeutic effects on AD [9–14], suggesting that diabetes and AD may share genetic etiological risk factors, especially provide a potential novel approach for AD drug development.

These studies imply that diabetes is closely associated with the risk of AD, and antidiabetic drugs also attracted much attention in the treatment of AD; however, it is unclear whether diabetes has causal associations with AD, and the impact of antidiabetic drug targets against AD remains to be further estimated. Mendelian randomization uses genetic variants as proxies for modifiable risk factors to test whether the risk factor is causally relevant to an outcome of interest, which could minimize the impact of confounding factors [15]. Thus, the present study performed a two-sample Mendelian randomization (2SMR) analysis to assess: i) whether diabetes is causally related to the increased risk of AD; ii) and if so, which diabetes-related physiological parameters, like blood glucose, insulin, and others, is associated with AD; iii) how diabetes drugs can be used as a candidate for the treatment of AD.

## Methods

Based on existing data sources of the MR-base platform, we selected genetic variants associated with the exposure measure as an instrument to estimate causal effects. Candidate genetic variants of outcome (AD) were obtained from the International Genomics of Alzheimer's Project (IGAP) [16]. As for exposures, we searched the EBI-GWAS database by the MR-base platform with the following terms: “type 1 diabetes” and “type 2 diabetes”. And 10 T1D-related SNPs were extracted from a European ancestry-specific joint GWA study to estimate the association between T1D and AD [17], while a total of 37 SNPs provided by the summary statistics of 48,286 cases and 250,671 controls were included to test the causal effect of T2D on AD [18]. Further, to investigate how diabetes affects the risk of AD, we also analyzed AD and diabetes-related parameters, including fasting blood glucose, total cholesterol levels, and insulin levels [19–21]. Data extraction and 2SMR analyses were automatically conducted using the software R and TwoSample MR package 0.5.0, and genome-wide significant (p-value < 5 × 10^−8^) was chosen for computational analysis [15]. We selected inverse variance weighting (IVW) as the main analytical method, and various 2SMR methods, including weighted median, weighted mode, and MR-Egger, were employed to improve the reliability of the causal inference. P-value < 0.05 was chosen as the discriminant criterion for the statistical significance of the 2SMR study. Besides, to ensure the robustness of results, leave-one-out sensitivity analysis was used to test whether there is an SNP that has an excessive impact on MR estimates. Heterogeneity and pleiotropy tests were implemented based on the code contained in the TwoSample MR package. Cochran’s Q statistics were used to explore the size of heterogeneity, and whether there is pleiotropy was decided by the intercept term of MR-Egger method.

Besides, inspired by the benefits of antidiabetic drugs for AD, we then performed a further 2SMR analysis for the causal associations between antidiabetic drug targets and AD risk to assess the therapeutic effects. Firstly, we searched the DrugBank database (http://www.drugbank.ca/) with the term “diabetes” to retrieve antidiabetic drugs and target genes [22]. Drugs or compounds that have been approved or were being developed for the treatment of diabetes were collected as available antidiabetic drugs. The information was extracted from each drug, including the name of antidiabetic drug, DrugBank ID, target gene, and target type. Secondly, using the TwoSample MR package, we identified target-related SNPs based on the GTEx eQTL catalog [23]. By using SNPs associated with antidiabetic drug target genes and without any linkage disequilibrium, we calculated MR estimates and did not define tissue types. Since the number of SNPs contained in each drug target was relatively small, a more liberal P-value threshold (p-value < 5 × 10^−5^) was used to filter available instrumental variables. In addition to the above four methods, we also added another MR method, wald ratio, which used a single instrumental variable to estimate the causal association.

Furthermore, based on the IGAP database, the threshold of p-value < 1 × 10^−5^ was used to screen susceptibility-associated SNPs of AD. The identified significant SNPs were mapped into related susceptibility genes according to the location of the SNPs on human chromosomes. We constructed network-based analyses by the Search Tool for the Retrieval of Interacting Genes (STRING) databases to investigate the protein–protein interaction (PPI) information between the identified targets and susceptibility genes [24], and the final network was visualized by Cytoscape software (Version 3.7.1) [25].

## Results

### Diabetes and AD

**Fig. 1.**
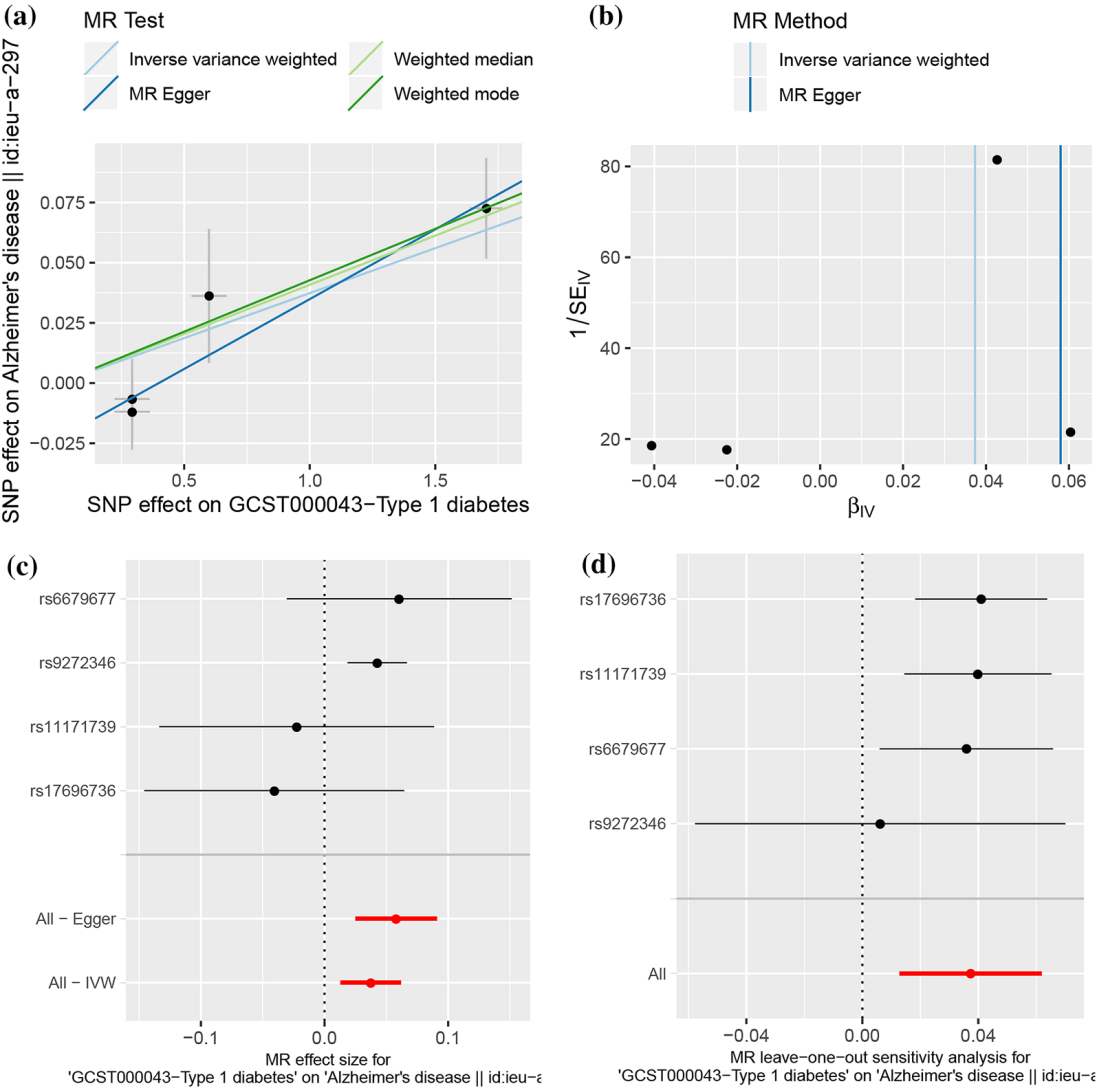
2SMR analysis of the causal association between T1D and the risk of AD. **a** Scatter plot. The slope of the line corresponds to a causal estimate using each of the four different methods. **b** Funnel plot. The vertical line shows a causal estimate using all SNPs combined into a single instrument for each of two different methods. **c** Forest plot. Each black dot represents the MR estimate of each SNP using the wald ratio, and the horizontal line represents the 95% CI. The red points show a combined causal estimate using all SNPs in a single instrument, including the 2SMR estimates of IVW and MR-Egger. **d** Leave-one-out sensitivity analysis. Each black dot represents the result of MR-IVW excluding that particular SNP, and the red dot depicts the IVW estimate using all SNPs

The 2SMR analysis provided a suggestive association between genetically predicted T1D and higher risks of AD (IVW, OR = 1.04, 95% CI = [1.01, 1.06], p = 2.90E-03, Table 1, Fig. 1). Cochran’s Q statistics showed little evidence of heterogeneity between T1D and AD, and the MR-Egger intercept suggested that there was no pleiotropy in the SNPs included in this study. The further leave-one-out analysis also found that there were no SNP had an excessive impact on the results (all lines are on the right side of 0). However, compared with other SNPs, the independent SNP rs9272346 exerted a relatively significant effect on the association between T1D and AD risk. According to the NCBI database, rs9272346 was located at HLA-DQA1, and the protein encoded by which plays a central role in the immune system by presenting peptides derived from extracellular proteins.

**Table 1:** 2SMR estimates of the causality between diabetes and AD

Study	Method	Number of SNPs	b	se	P-value	OR	95% CI	Cochran’s Q statistic (P-value)	MR-egger intercept (P-value)
T1D	Inverse variance weighted	4	0.04	0.01	2.90E−03	1.04	1.01–1.06	3.66 (0.30)	
	MR Egger	4	0.06	0.02	0.08	1.06	1.03–1.10	0.94 (0.63)	− 0.02 (0.24)
	Weighted median	4	0.04	0.01	8.83E−04	1.04	1.02–1.07		
	Weighted mode	4	0.04	0.01	0.04	1.04	1.02–1.07		
T2D	Inverse variance weighted	11	0.29	0.12	0.02	1.34	1.05–1.70	9.43 (0.49)	
	MR Egger	11	0.70	0.40	0.11	2.01	0.93–4.36	8.28 (0.51)	− 0.02 (0.31)
	Weighted median	11	0.18	0.17	0.28	1.20	0.86–1.68		
	Weighted mode	11	0.04	0.23	0.88	1.04	0.65–1.64

**Fig. 2.**
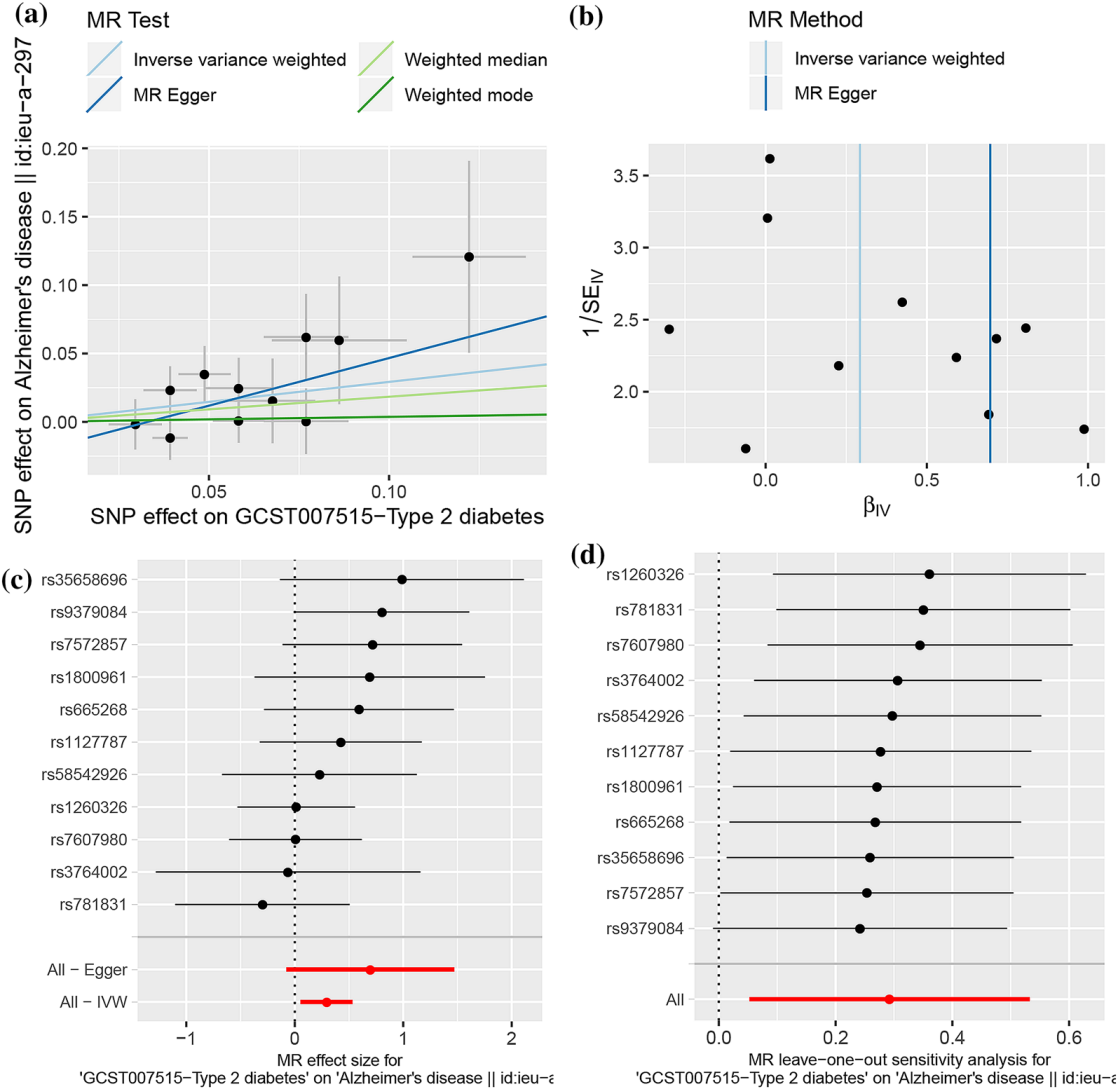
2SMR analysis of the causal association between T2D and the risk of AD. **a** Scatter plot. The slope of the line corresponds to a causal estimate using each of the four different methods. **b** Funnel plot. The vertical line shows a causal estimate using all SNPs combined into a single instrument for each of two different methods. **c** Forest plot. Each black dot represents the MR estimate of each SNP using the wald ratio, and the horizontal line represents the 95% CI. The red points show a combined causal estimate using all SNPs in a single instrument, including the 2SMR estimates of IVW and MR-Egger. **d** Leave-one-out sensitivity analysis. Each black dot represents the result of MR-IVW excluding that particular SNP, and the red dot depicts the IVW estimate using all SNPs

Compared with T1D, T2D seemed to show a much stronger association with an increased risk of AD (IVW, OR = 1.34, 95% CI = [1.05, 1.70], p = 0.02, Fig. 2). Cochran’s Q statistics showed little evidence of heterogeneity between T2D and AD. The MR-Egger intercept suggested that there was no pleiotropy in the SNPs included in this study. Moreover, the leave-one-out method did not find that a certain SNP would have an excessive impact on the MR results, which also supported that the MR results were robust.

### Diabetes-related parameters and AD

**Fig. 3.**
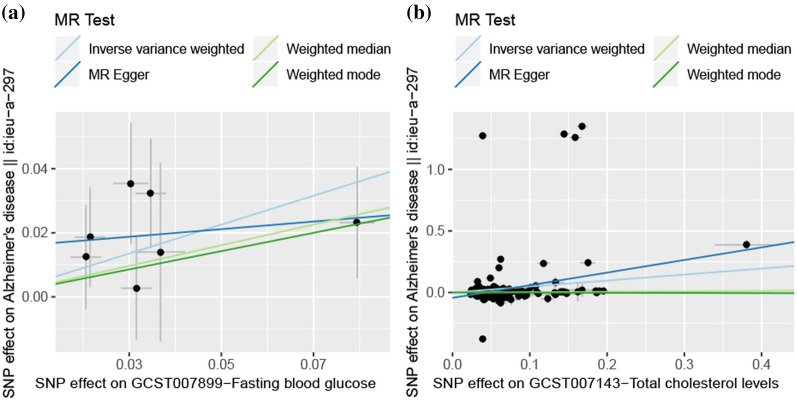
2SMR estimates of the causality between fasting blood glucose and total cholesterol levels and AD. a) the causal effects of fasting blood glucose and AD. b) the causal effects of total cholesterol levels and AD

In addition, we also conducted further analysis to investigate the causal association between diabetes-related physiological parameters and the risk of AD. In view of the fact that blood glucose and dyslipidemia are widely recognized as physiological changes in diabetes, we conducted a 2SMR analysis to evaluate their causal association with AD. By performing a 2SMR analysis of the diabetes-related physiological parameters and AD, we found that fasting blood glucose and total cholesterol levels may have a causative role in the development of AD as shown in Fig. 3. Fasting blood glucose was associated with a 57% increase in the risk of AD (IVW, OR = 1.57, 95% CI = [1.14, 2.17], p = 6.33E-03), total cholesterol levels also showed a strong causal association with the risk of AD (IVW, OR = 1.62, 95% CI = [1.21, 2.18], p = 1.23E-03). Besides, as one of the typical characteristics of diabetes, the causal association between insulin level and AD was also included in this study. However, based on the currently available data, the 2SMR analysis results did not support the causal effect of insulin levels on AD risk (data not shown).

### Antidiabetic drugs and AD

Based on the DrugBank database, we obtained 74 antidiabetic drugs up to July 2021, covering 96 target and enzyme genes extracted from the involved drugs. The details of these drugs, including drug names, DrugBank ID, target genes, and enzyme genes, are displayed in Table 2.

**Table 2:** Main characteristics of the antidiabetic drugs included in the PPI network

Name	Drugbank ID	Target genes	Target type
Ebselen	DB12610	EPHX2	Target
INCB13739	DB05064	HSD11B1	Target
PSN357	DB05044	PYGL	Target
Bisegliptin	DB06127	DPP4	Target
NOX-700	DB05464	NFKB2	Target
		NFKB1	Target
CLX-0921	DB05854	PPARG	Target
Reglitazar	DB04971	PPARA	Target
		PPARG	Target
ISIS 113715	DB05506	PTPN1	Target
AT1391	DB05120	INSR	Target
NN344	DB05115	INSR	Target
		CYP1A2	Enzyme
APD668	DB05166	GPR119	Target
Dutogliptin	DB11723	DPP4	Target
MB-07803	DB05053	FBP1	Target
PSN9301	DB05001	DPP4	Target
Gliquidone	DB01251	ABCC8	Target
		KCNJ8	Target
		CYP2C9	Enzyme
Albiglutide	DB09043	GLP1R	Target
Pramlintide	DB01278	CALCR	Target
		RAMP1	Target
		RAMP2	Target
		RAMP3	Target
Voglibose	DB04878	MGAM	Target
Dapagliflozin	DB06292	SLC5A2	Target
		CYP1A1	Enzyme
		CYP1A2	Enzyme
		CYP2A6	Enzyme
		CYP2C9	Enzyme
		CYP2D6	Enzyme
		CYP3A4	Enzyme
		UGT1A9	Enzyme
		UGT2B4	Enzyme
		UGT2B7	Enzyme
Miglitol	DB00491	MGAM	Target
		GAA	Target
		GANAB	Target
		GANC	Target
		AMY2A	Enzyme
Vildagliptin	DB04876	DPP4	Target
Dulaglutide	DB09045	GLP1R	Target
Phenformin	DB00914	PRKAA1	Target
		KCNJ8	Target
		CYP2D6	Enzyme
AMG-131	DB05490	PPARG	Target
Acarbose	DB00284	MGAM	Target
		GAA	Target
		SI	Target
		AMY2A	Target
Sitagliptin	DB01261	DPP4	Target
		CYP3A4	Enzyme
		CYP2C8	Enzyme
Acetohexamide	DB00414	KCNJ1	Target
		CBR1	Enzyme
		CYP2C9	Enzyme
Canagliflozin	DB08907	SLC5A2	Target
		UGT1A9	Enzyme
		UGT2B4	Enzyme
		CYP3A4	Enzyme
Pioglitazone	DB01132	PPARG	Target
		MAOB	Target
		CYP2C8	Enzyme
		CYP3A4	Enzyme
		CYP1A1	Enzyme
Glisoxepide	DB01289	KCNJ8	Target
		CYP2C9	Enzyme
Glipizide	DB01067	ABCC8	Target
		PPARG	Target
		CYP2C9	Enzyme
		UGT1A1	Enzyme
Insulin Glargine	DB00047	INSR	Target
		IGF1R	Target
		CYP1A2	Enzyme
Insulin Degludec	DB09564	INSR	Target
		IGF1R	Target
		CYP1A2	Enzyme
Chlorpropamide	DB00672	ABCC8	Target
		CYP2C9	Enzyme
		CYP2C19	Enzyme
		PTGS1	Enzyme
Linagliptin	DB08882	DPP4	Target
		CYP3A4	Enzyme
Repaglinide	DB00912	ABCC8	Target
		PPARG	Target
		CYP2C8	Enzyme
		CYP3A4	Enzyme
Insulin Pork	DB00071	INSR	Target
		IGF1R	Target
		IDE	Enzyme
		CYP1A2	Enzyme
Nateglinide	DB00731	ABCC8	Target
		PPARG	Target
		CYP2C9	Enzyme
		CYP3A4	Enzyme
		CYP3A5	Enzyme
		CYP3A7	Enzyme
		PTGS1	Enzyme
		UGT1A9	Enzyme
		CYP2D6	Enzyme
Insulin Aspart	DB01306	INSR	Target
		IGF1R	Target
		CYP1A2	Enzyme
Insulin Detemir	DB01307	INSR	Target
		IGF1R	Target
		CYP1A2	Enzyme
Saxagliptin	DB06335	DPP4	Target
		CYP3A4	Enzyme
		CYP3A5	Enzyme
Insulin Glulisine	DB01309	INSR	Target
		IGF1R	Target
		CYP1A2	Enzyme
Tolbutamide	DB01124	ABCC8	Target
		KCNJ1	Target
		CYP2C9	Enzyme
		CYP2C8	Enzyme
		CYP2C19	Enzyme
		CYP2C18	Enzyme
Rosiglitazone	DB00412	PPARG	Target
		ACSL4	Target
		PPARA	Target
		PPARD	Target
		RXRA	Target
		RXRB	Target
		RXRG	Target
		CYP2C8	Enzyme
		CYP2C9	Enzyme
		PTGS1	Enzyme
		CYP1A2	Enzyme
		CYP3A4	Enzyme
		CYP2B6	Enzyme
		CYP2D6	Enzyme
		CYP2E1	Enzyme
Mitiglinide	DB01252	ABCC8	Target
		PPARG	Target
		UGT1A3	Enzyme
		UGT2B7	Enzyme
Insulin Human	DB00030	INSR	Target
		IGF1R	Target
		CPE	Target
		NOV	Target
		LRP2	Target
		IGFBP7	Target
		IDE	Enzyme
		PCSK2	Enzyme
		PCSK1	Enzyme
		CYP1A2	Enzyme
Insulin Lispro	DB00046	INSR	Target
		IGF1R	Target
		CYP1A2	Enzyme
		IDE	Enzyme
Lixisenatide	DB09265	GLP1R	Target
Metformin	DB00331	PRKAB1	Target
		ETFDH	Target
		GPD1	Target
Lobeglitazone	DB09198	PPARG	Target
		CYP1A2	Enzyme
		CYP2C9	Enzyme
		CYP2C19	Enzyme
		CYP3A4	Enzyme
Managlinat dialanetil	DB05518	FBP1	Target
Levoketoconazole	DB05667	CYP11B1	Target
		CYP51A1	Target
		CYP3A4	Enzyme
		CYP3A5	Enzyme
		CYP51A1	Enzyme
		CYP17A1	Enzyme
		CYP21A2	Enzyme
		CYP11B1	Enzyme
Tesaglitazar	DB06536	PPARA	Target
		PPARG	Target
Ertiprotafib	DB06521	PTPN1	Target
		IKBKB	Target
		PPARA	Target
		PPARG	Target
Glycodiazine	DB01382	KCNJ1	Target
		ABCC8	Target
Muraglitazar	DB06510	PPARA	Target
		PPARG	Target
		CYP1A2	Enzyme
		UGT1A3	Enzyme
		UGT1A1	Enzyme
		CYP2C8	Enzyme
Troglitazone	DB00197	PPARG	Target
		ACSL4	Target
		SERPINE1	Target
		SLC29A1	Target
		ESRRG	Target
		ESRRA	Target
		PPARD	Target
		PPARA	Target
		GSTP1	Target
		CYP3A4	Enzyme
		CYP2C19	Enzyme
		UGT1A1	Enzyme
		CYP2C8	Enzyme
		CYP19A1	Enzyme
		CYP1A1	Enzyme
		CYP2B6	Enzyme
		CYP2C9	Enzyme
		CYP3A5	Enzyme
		CYP3A7	Enzyme
		UGT1A3	Enzyme
		UGT1A4	Enzyme
		UGT1A6	Enzyme
		UGT1A7	Enzyme
		UGT1A8	Enzyme
		UGT1A9	Enzyme
		UGT1A10	Enzyme
		UGT2B7	Enzyme
		UGT2B15	Enzyme
Ertugliflozin	DB11827	SLC5A2	Target
		UGT1A9	Enzyme
		UGT2B7	Enzyme
		UGT1A1	Enzyme
		UGT1A4	Enzyme
Exenatide	DB01276	GLP1R	Target
		DPP4	Enzyme
Naveglitazar	DB12662	PPARG	Target
Alogliptin	DB06203	DPP4	Target
		CYP3A4	Enzyme
		CYP2D6	Enzyme
Liraglutide	DB06655	GLP1R	Target
		DPP4	Enzyme
		MME	Enzyme
Semaglutide	DB13928	GLP1R	Target
		DPP4	Enzyme
		MME	Enzyme
		LPL	Enzyme
		AMY1A	Enzyme
Glimepiride	DB00222	KCNJ11	Target
		KCNJ1	Target
		ABCC8	Target
		CYP2C9	Enzyme
Sarpogrelate	DB12163	HTR2C	Target
		HTR2A	Target
Glyburide	DB01016	ABCC9	Target
		ABCB11	Target
		ABCA1	Target
		CFTR	Target
		CPT1A	Target
		TRPM4	Target
		CYP2C9	Enzyme
		CYP2C19	Enzyme
		CYP3A4	Enzyme
		CYP3A7	Enzyme
		CYP3A5	Enzyme
Gliclazide	DB01120	ABCC8	Target
		VEGFA	Target
		CYP2C9	Enzyme
		CYP2C19	Enzyme
Empagliflozin	DB09038	SLC5A2	Target
		UGT2B7	Enzyme
		UGT1A3	Enzyme
		UGT1A8	Enzyme
		UGT1A9	Enzyme
Glymidine	DB01382	KCNJ1	Target
		ABCC8	Target
Balaglitazone	DB12781	CYP3A4	Enzyme
		CYP2C8	Enzyme
Glibornuride	DB08962	CYP2C9	Enzyme
Rivoglitazone	DB09200	CYP3A4	Enzyme
		CYP2C8	Enzyme
AB192	DB06111	MPO	Enzyme
Lisofylline	DB12406	CYP1A2	Enzyme

**Fig. 4.**
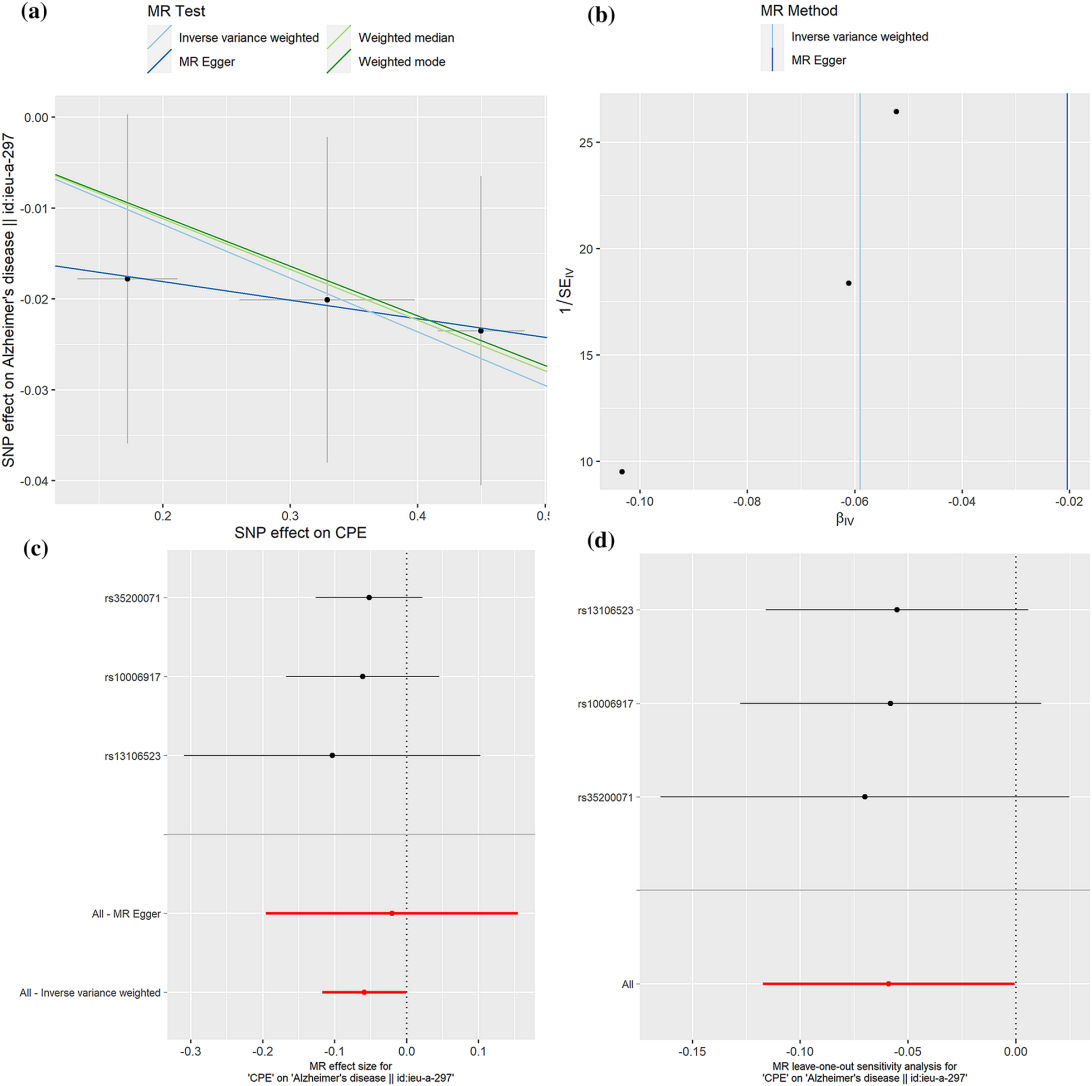
2SMR estimates of the causality between CPE target and AD. **a** Scatter plot. The slope of the line corresponds to a causal estimate using each of the four different methods. **b** Funnel plot. The vertical line shows a causal estimate using all SNPs combined into a single instrument for each of two different methods. **c** Forest plot. Each black dot represents the MR estimate of each SNP using the wald ratio, and the horizontal line represents the 95% CI. The red points show a combined causal estimate using all SNPs in a single instrument, including the 2SMR estimates of IVW and MR-Egger. **d** Leave-one-out sensitivity analysis. Each black dot represents the result of MR-IVW excluding that particular SNP, and the red dot depicts the IVW estimate using all SNPs

**Fig. 5.**
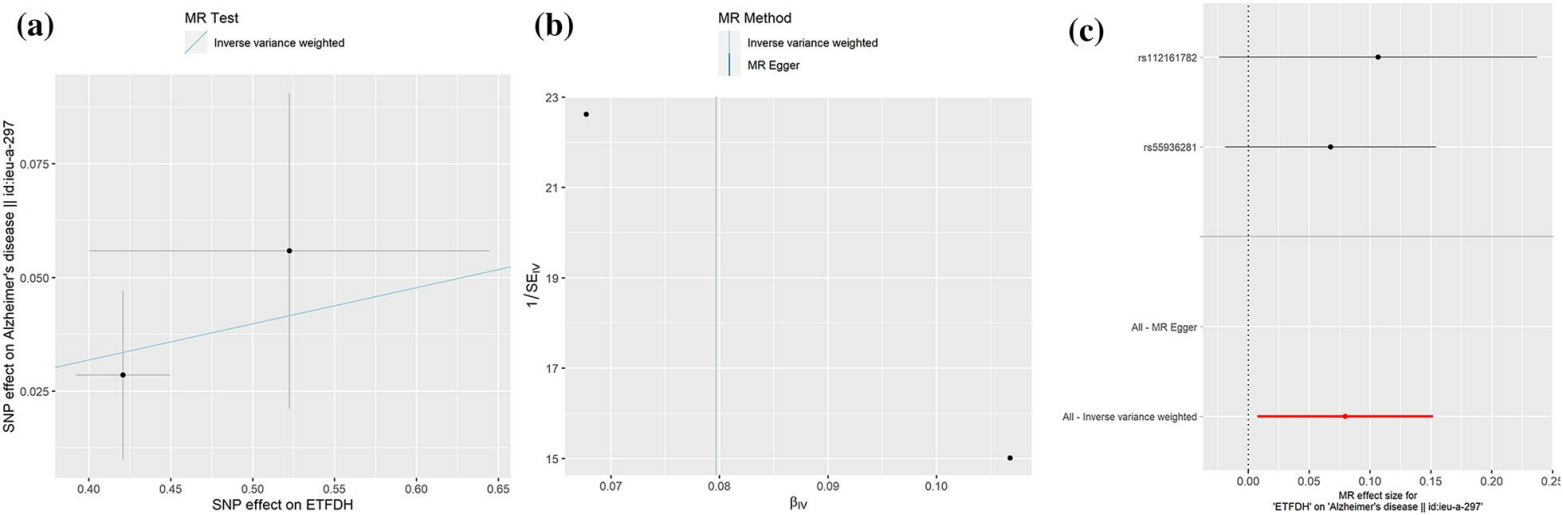
2SMR estimates of the causality between ETFDH target and AD. **a** Scatter plot. The slope of the line corresponds to a causal estimate using each of the four different methods. **b** Funnel plot. The vertical line shows a causal estimate using all SNPs combined into a single instrument for each of two different methods. **c** Forest plot. Each black dot represents the MR estimate of each SNP using the wald ratio, and the horizontal line represents the 95% CI. The red points show a combined causal estimate using all SNPs in a single instrument, including the 2SMR estimates of IVW and MR-Egger

**Fig. 6.**
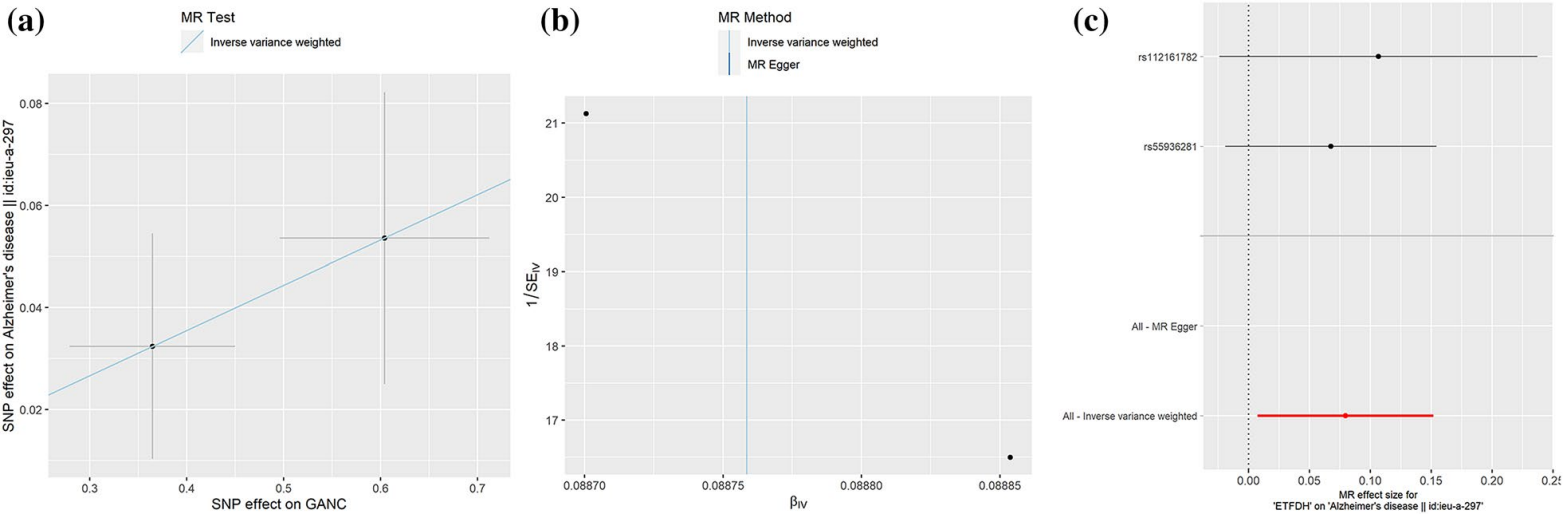
2SMR estimates of the causality between GANC target and AD. **a** Scatter plot. The slope of the line corresponds to a causal estimate using each of the four different methods. **b** Funnel plot. The vertical line shows a causal estimate using all SNPs combined into a single instrument for each of two different methods. **c** Forest plot. Each black dot represents the MR estimate of each SNP using the wald ratio, and the horizontal line represents the 95% CI. The red points show a combined causal estimate using all SNPs in a single instrument, including the 2SMR estimates of IVW and MR-Egger

By using SNPs associated with antidiabetic drug target genes (p-value < 5 × 10^−5^) as instrumental variables, we conducted a 2SMR analysis for the causal associations between antidiabetic drug targets and AD risk (Table 3). Preliminary results showed that four targets, including carboxypeptidase E (CPE), electron transfer flavoprotein-ubiquinone oxidoreductase (ETFDH), neutral alpha-glucosidase C (GANC), and maltase-glucoamylase (MGAM), were identified to be causally associated with AD. Among them, genetically predicted the CPE gene could be a protective factor in AD (IVW, OR = 0.94, 95%CI = [0.89, 1.00], p = 0.05, Fig. 4), while the expressions of ETFDH (IVW, OR = 1.08, 95%CI = [1.01,1.16], p = 0.03, Fig. 5), GANC (IVW, OR = 1.09, 95%CI = [1.02,1.18], p = 0.02, Fig. 6), and MGAM (Wald ratio, OR = 1.04, 95%CI = [1.00,1.09], p = 0.04) were causally associated with the increased risk of AD. Notably, the present study showed high expressions of ETFDH, GANC, and MGAM have causal effects on the increased risk of AD, in other words, inhibiting the expression of three target genes is beneficial to the treatment of AD to a certain extent. Interestingly, based on the pharmacological actions obtained from the DrugBank database, three targets related to antidiabetic drugs, including metformin, miglitol, acarbose, voglibose, were the corresponding inhibitors of the above targets, suggesting that identified targets might provide useful genetic clues to understand the anti-AD effects of selected antidiabetic drugs.

**Table 3:** 2SMR estimates of the causality between antidiabetic targets and AD

Target gene	Drugs	Action	Method	Numbers of SNPs	OR	95% CI	P-value
CPE	Insulin Human	Modulator (Unknown)	MR Egger	3	0.98	0.82–1.17	0.86
			Inverse variance weighted	3	0.94	0.89–1.00	0.05
			Weighted median	3	0.95	0.89–1.01	0.08
			Weighted mode	3	0.95	0.89–1.01	0.24
ETFDH	Metformin	Inhibitor	Inverse variance weighted	2	1.08	1.01–1.16	0.03
GANC	Miglitol	Antagonist	Inverse variance weighted	2	1.09	1.02–1.18	0.02
MGAM	Voglibose	Inhibitor	Wald ratio	1	1.04	1.00–1.09	0.04
	Acarbose	Inhibitor					
	Miglitol	Antagonist, inhibitor

**Fig. 7:**
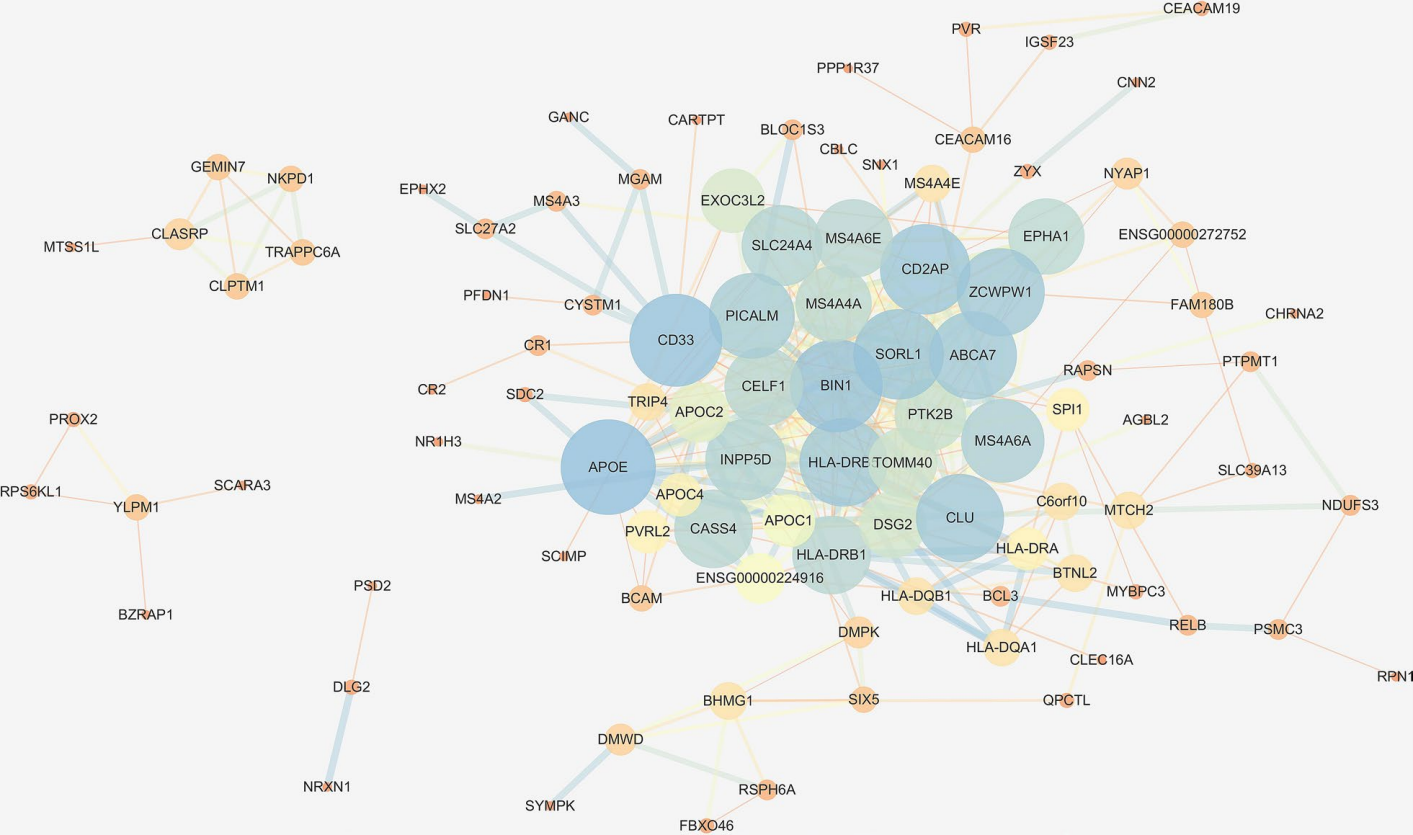
A network-based analysis based on identified targets (CPE, ETFDH, GANC, MGAM) and AD susceptibility genes. The combined score is mapped to the edge size (low values to small sizes and bright color), and the node degree is mapped to the node size and node color (low values to small sizes and bright color)

**Fig. 8:**
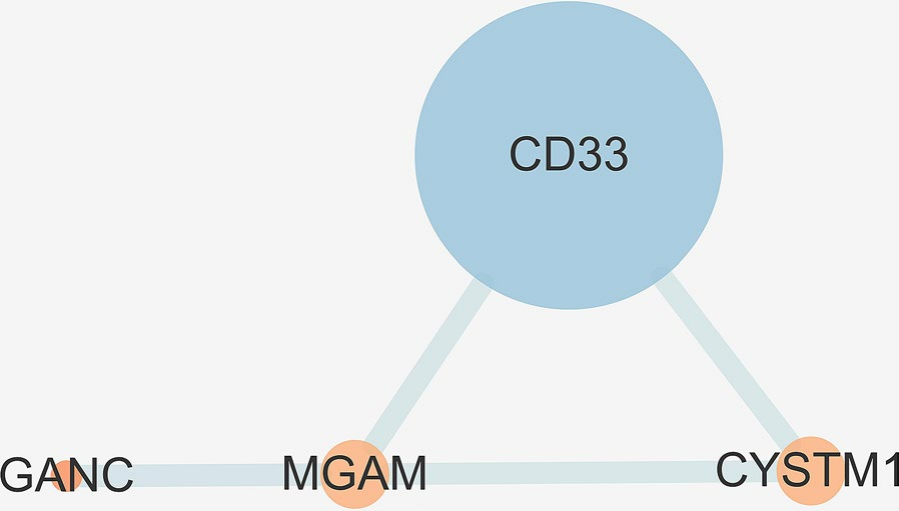
The sub-network analysis based on identified targets and first neighbors of AD susceptibility genes

Furthermore, a total of 2746 SNPs of AD were discovered from the IGAP database using a genome-wide significance threshold (p-value < 1 × 10^−5^). By mapping the significant SNPs to genes on the basis of the NCBI database, 152 AD susceptibility genes were identified and included in this study. A PPI network that followed was constructed by identified targets (CPE, ETFDH, GANC, MGAM) and AD susceptibility genes (Fig. 7). It was found that CPE and ETFDH were not interacted with any degree in the network, while GANC was related to MGAM, and further interacted with CD33 (Fig. 8), which was a strong genetic locus associated with AD.

## Discussion

Through performing a 2SMR analysis of the available data, we found that diabetes had a causal effect on AD risk, which is in line with previous epidemiological studies. There should be multiple mechanisms underlying the association between diabetes and AD. First, insulin signaling dysregulation may be a critical pathological change in AD, and it has been reported that insulin signaling is impaired in postmortem brain tissue from AD patients [26, 27]. The insulin signaling pathway contributes to the control of neuronal excitability and metabolism, and cerebrovascular changes, such as inflammation and alterations in brain insulin signaling, might play a pivotal role in AD development [28, 29]. Second, as a mechanistic linker between AD and diabetes, inflammation can accelerate the development of diabetes by influencing islet function and peripheral insulin sensitivity. Moreover, as a starting point of AD pathological progression, the normal synaptic function will be disrupted by cerebrovascular and central inflammation, along with the increased accumulation of Aβ [30].

Further 2SMR analysis revealed that some diabetes-related physiological parameters, such as fasting blood glucose and total cholesterol levels, were causally associated with the risk of AD. Previous studies have demonstrated that metabolic dysfunction of diabetes, especially glucose-related dysfunction, may play a causative role in the development of AD. For example, a large-scale genome-wide cross-trait analysis identified 4 loci that were associated with AD and fasting glucose [31]. Also, as the most cholesterol-rich organ, the cholesterol homeostasis in the human brain may be closely related to the occurrence and development of AD [32]. Recent studies have indicated that lipid metabolism-related genes, such as APOC1 and APOE, might be major risk factors for AD due to the involvement in the maintenance of brain lipid homeostasis [33, 34]. Furthermore, our previous study also identified a total of six SNPs shared between T2D and AD and found that lipid metabolism-related pathways were common between the two disorders by functional enrichment analysis [35].

In the past decades, theoretical and experimental investigations of novel drugs for AD have attracted much attention. It is noteworthy that drug repositioning based on the approved drugs may represent an important source for AD drug discovery, a case of this is antidiabetic drug repositioning. By the 2SMR analysis, four targets, including CPE, ETFDH, GANC, and MGAM, were identified to be causally associated with AD in this paper. In particular, in combination with the present 2SMR results and pharmacological actions obtained from the DrugBank database, ETFDH-, GANC-, and MGAM-related antidiabetic drugs, including metformin, miglitol, acarbose, voglibose, were precisely the corresponding inhibitors of the above targets, indicating potential therapeutic effects on AD. Notably, among those, miglitol, acarbose, and voglibose are currently used in the management of glycemic control by inhibiting α-glucosidase, which is an important biological target/enzyme that can catalyze the degradation of dietary polysaccharides into monosaccharides. The preliminary data in this paper proposed that the targets of α-glucosidase inhibitors, for example, GANC and MGAM, were causally associated with the increased risk of AD, suggesting the therapeutic implications of α-glucosidase on AD. However, at present, the antidiabetic drugs for the treatment of AD mainly focus on GLP-1R agonists (liraglutide, exenatide), thiazolidinediones (pioglitazone, rosiglitazone), DPP-4 inhibitors (sitagliptin, vildagliptin), and so on, while there are limited studies of α-glucosidase inhibitors in the treatment of AD, and these findings remain to be further estimated.

Several limitations of the present analysis need to be noted. In the 2SMR analysis, we avoided the influence of different ethnicities to the greatest extent by screening for European ancestry in the involved studies. However, there are also a few studies that have mixed populations with a small proportion outside Europe. At the same time, the limitation of European ancestry also indicates that our findings may not be applicable to other ethnicities. In addition, the small number of variants for each exposure is the limitation of these analyses. These factors may interfere with the stability of the conclusion.

## Conclusions

The present 2SMR analysis based on extensive data uncovered causal associations between diabetes and AD. It is interesting to note that T2D seemed to show a more significant association with AD risk than T1D. Further analysis identified several diabetes-related physiological parameters that may have a causative role in the development of AD. Besides, four targets from antidiabetic drugs were identified to be causally associated with AD, indicating potential therapeutic effects on AD and might provide implications for drug development. In summary, our study indicates that diabetes and antidiabetic drugs were causally relevant to AD and certainly warrants more well-designed studies clinical verifications in the future. At the same time, these findings also inspire us that preventing or delaying the risk factors of AD, such as diabetes, are likely to be more achievable goals in the foreseeable future.

## Data Availability

The data is available from the corresponding author upon request.

## Abbreviations

AD: Alzheimer’s disease
T1D: Type 1 diabetes
T2D: Type 2 diabetes
2SMR: Two-sample Mendelian randomization
IGAP: International genomics of Alzheimer's project
SNPs: Single nucleotide polymorphisms
